# Mixed influence of COVID-19 on primary maternal and child health services in sub-Saharan Africa: a scoping review

**DOI:** 10.3389/fpubh.2024.1399398

**Published:** 2024-06-24

**Authors:** Bienvenu Salim Camara, Alison M. El Ayadi, Appolinaire S. Thea, Fatoumata B. Traoré, El Hadj M. Diallo, Mathias Doré, Jean-Baptiste D. Loua, Mabinty Toure, Alexandre Delamou

**Affiliations:** ^1^Africa Center of Excellence for Prevention and Control of Communicable Diseases (CEA-PCMT), Gamal Abdel Nasser University of Conakry, Conakry, Guinea; ^2^Department of Public Health, Gamal Abdel Nasser University of Conakry, Conakry, Guinea; ^3^Centre National de Formation et de Recherche en Santé Rurale de Maferinyah, Forécariah, Guinea; ^4^Department of Obstetrics, Gynecology and Reproductive Sciences, Bixby Center for Global Reproductive Health, University of California, San Francisco, San Francisco, CA, United States; ^5^National Institute of Public Health, Bamako, Mali

**Keywords:** primary healthcare, maternal and child health, COVID-19, resilience, health systems, Sub-Saharan Africa, scoping review

## Abstract

**Introduction:**

The COVID-19 pandemic profoundly affected the provision of and demand for routine health services in the world. The objective of this scoping review was to synthesize the influence of the COVID-19 pandemic on primary maternal and child health (MCH) services in sub-Saharan Africa.

**Methods:**

The studies searched original studies reporting on the influence of the COVID-19 pandemic on primary MCH services. Four scientific databases (Pubmed, AJOL, CAIRN, CINAHL) and one gray literature database (Google Scholar) were used for this search. We also searched through the snowball citation approach and study reference lists.

**Results:**

The influence of the COVID-19 pandemic on primary MCH services has been mixed in sub-Saharan Africa. Attendance at some health centers declined for antenatal care, deliveries, immunization, and pneumonia cases. Other health centers did not experience a significant influence of the pandemic on some of these services. In fact, antenatal care increased in a number of health centers. MCH service indicators which declined during COVID-19 were linked on the demand side to regulatory measures against COVID-19, the perceived unavailability of resources for routine services, the perceived negative attitude of staff in these facilities, the perceived transmission risk in primary health care facilities and the perceived anticipated stigma. On the supply side, factors included the lack of equipment in primary facilities, the lack of guidelines for providing care in the pandemic context, the regulatory measures against COVID-19 taken in these facilities, and the lack of motivation of providers working in these facilities.

**Conclusion:**

This study recommends prioritizing the improvement of infection prevention measures in primary health care facilities for resilience of MCH indicators to epidemic crises. Improvement efforts should be tailored to the disparities in preventive measures between health centers. The identification of best practices from more resilient health centers could better guide these efforts.

## 1 Introduction

The 2019 coronavirus (COVID-19) pandemic is one of the largest and most lethal epidemics in history and has challenged health systems worldwide (1). In fact, in several countries, health system resilience prioritized mitigating the spread and direct consequences of COVID-19 over ensuring the continuity of essential routine health services (2, 3). A global review covering 106 countries showed that only 34% of countries had made reference to maintaining essential routine health services as a priority within their resilience plans for the COVID-19 pandemic, and that only 24% had a budget line dedicated to maintaining these services (2). Consequently, the pandemic has profoundly affected the provision of and demand for routine health services in most countries (1, 4). A review of 81 studies involving more than 17 million health services provided across 20 countries reported a median 37% reduction in health service use in 2020 compared to the previous year (5). This resulted in increases in cases and deaths related to other diseases during the COVID-19 period (4, 5).

Routine health services in developing countries might have experienced an even greater disruption by the COVID-19 pandemic due to populations' poor socio-economic status and less resilient health systems (6). For example, unlike developed countries (7), many of these health systems on the one hand and populations on the other hand could lack appropriate telehealth technology services, hindering patients' abilities to use these services to access care without attending a health facility. The implementation and application of effective strategies to mitigate COVID-19's impact on services in health facilities constituted another challenge limiting the capacity of these health systems; for example, the lack of suitable infrastructure would compromise compliance with physical distancing, just as the lack of personal protective equipment or rapid tests for COVID-19 affected the optimal provision of health services (8).

In such context, maternal and child health (MCH) services would be more compromised because of the risk of transmission involved in providing these services (9). Firstly, most of them being provided as routine services (antenatal visits, vaccination), they are characterized by a plethora of patients in the care units; the risk of such massive gathering could frighten users and even some providers from attending these units. Furthermore, some of the physiological signs of pregnancy overlap with those of COVID-19, including coughing and breathing difficulties. Similarly, common symptoms of childhood illnesses such as fever, cough, and cold overlap with the signs of COVID-19. Consequently, children and pregnant women presenting with these symptoms could be denied health services because of fear for contamination of health staffs or other patients; as well on the demand side, caregivers and pregnant women could refrain from attending health facilities for such symptoms.

Declining use of MCH services in sub-Saharan Africa (SSA) has been reported by a few reviews (10–12) indicating significant drops in antenatal care (ANC) visits, facility-based deliveries and childhood vaccination. Reported reasons for these drops include inappropriate service delivery, pandemic preventive measures, shortage of medical supplies, staff workload, fear of COVID-19 infection and transport inaccessibility (11).

Although very informative, these reviews do not report on postpartum care or service use for the most common life-threatening illnesses affecting children in SSA such as malaria, pneumonia, and diarrhea (13, 14). Understanding as well how these essential services were affected by the COVID-19 pandemic may inform strategies for resilience to maintain such services during the future epidemics. Furthermore, the key indicators for MCH services as synthesized by these reviews combine indicators for services provided at the primary, secondary and (probably) tertiary levels, masking the impact at each distinct level of the healthcare system. Yet, such disaggregation could better guide interventions for health system resilience in the face of possible future epidemic crises. Therefore, synthesizing evidence on the influence of the COVID-19 pandemic on primary MCH services alone in SSA could help to better guide health system resilience policies for optimal access of women and children to essential health care in times of epidemic crises. Thus, the objective of this scoping review was to synthesize research findings on the influence of the COVID-19 pandemic on primary MCH services in SSA. Specifically, we sought to summarize the effect of the pandemic on MCH indicators as well as the factors that explain any change in these indicators.

## 2 Materials and methods

Colquhoun et al. defined a scoping review as a form of knowledge synthesis that responds to an exploratory question, aiming to map key concepts, types of evidence and gaps in related research by systematically searching, selecting and synthesizing existing knowledge (15). Given the need to synthesize not only measures of the effect of the pandemic on MCH indicators but also the experiences that have led to this effect, a scoping review is the type of review best suited to achieving the objectives of this research. This scoping review followed the steps established by Arksey and O'Malley (16) and Levac et al. (17), and was reported following to the Preferred Reporting Items for Systematic Reviews and Meta-Analyses (PRISMA) checklist (18).

### 2.1 Identification of the research questions

To achieve the objectives of this review, the following research questions were targeted:

1. What is the influence of the COVID-19 pandemic on the use of primary MCH services in SSA?
2. What COVID-19-related explanatory factors were investigated and how did they influence on the use of primary MCH services in SSA?

In this scoping review, we sought to map the primary MCH service indicators that were vulnerable to the COVID-19 pandemic in SSA and to understand how COVID-19 influenced these indicators. We also sought to identify areas that require additional research on the influence of the COVID-19 pandemic on primary MCH services in SSA.

### 2.2 Identification of relevant studies

We searched for various types of studies for this review, including systematic reviews, qualitative and quantitative studies that report on the influence of the COVID-19 pandemic on primary MCH services in SSA within the following scientific literature databases: Pubmed, African Journals Online, the Journals and Works in the Humanities and Social Sciences (CAIRN), Cumulative Index to Nursing and Allied Health Literature (CINAHL). Gray literature was also used through a Google Scholar search. The most recent search was performed on March 12, 2023.

The search strategy combined five key concepts: maternal health service, child health services, COVID-19 pandemic, effect, SSA. We developed our search strategy through identifying relevant keywords and their synonyms. To identify keywords, we conducted a preliminary search on PubMed to identify some key studies on our research topic. From these studies, we extracted keywords and MeSH terms from the titles, abstracts, and keywords provided by the authors to develop a comprehensive list of keywords that informed our study identification process. Boolean operators were applied by combining key concepts in English and their synonyms including those with controlled vocabulary (MeSH terms), e.g., (“maternal health service” OR “maternal care pattern” OR “child health service” OR “neonatal care”) AND (“COVID-19 pandemic” OR “coronavirus pandemic”) AND (“sub-saharan Africa” OR Africa). The different article search strategies that were applied to the selected literature databases, respectively, can be found in Appendix A. The article search strategy was developed in PubMed and adapted to other databases. In addition, a snowball search was conducted by examining the reference lists of the included studies to find other relevant studies.

### 2.3 Study selection

Following the PRISMA flow diagram (19), the process of studies' selection consisted of identification, screening, eligibility check and inclusion. In the identification phase, all literature sources identified in the various scientific/academic and gray databases were combined and then duplicates were removed. In the screening phase, titles and abstracts of studies were examined; studies not relevant to the topic of our review were excluded. For eligibility check, the full text of each screened study was read; and eligibility was assessed based on the “population,” “concept” and “context” elements for scoping reviews as proposed by Joanna Briggs Institute (17). For this study, population included pregnant or postpartum women (first 6 weeks after delivery) and/or children under 5 years of age, residing in any of the SSA countries; the concept related to all of the following concepts: Maternal Health Service and/or Child Health Service, COVID-19 Pandemic, Influence; the context was SSA, as defined by the United Nations Development Programme, including 46 countries (Table 1). A study was deemed eligible when it met the eligibility criteria for the three elements. The screening and eligibility check were carried out in duplicate by BSC and DLJB. In both phases (screening and full text eligibility assessment), discrepancies between the two authors were discussed and resolved.

**Table 1:** Elements and description of the article selection criteria.

**Elements**	**Selection criteria**
Population	Included pregnant or postpartum women (first 6 weeks after delivery) and/or children under 5 years of age, residing in any of the sub-Saharan African countries.
Concept	Related to all of the following concepts: Maternal Health Service and/or Child Health Service, COVID-19 Pandemic, Influence. °*Maternal health services*: routine health system services provided to pregnant or postpartum women by community health workers or a primary health facility (health post, health center, or private clinic offering equivalent services considered by the study authors). °*Child health services*: health services offered to children under 5 years of age by community health workers or a primary health facility (health post, health center, or private clinic offering equivalent services considered by the study authors). These services include neonatal care, immunization, and screening and treatment of common childhood diseases in sub-Saharan Africa (malaria, respiratory infections, diarrheal diseases, etc.). °*COVID-19 Pandemic*: when the study referred to the Coronavirus pandemic. °*Influence*: influence of the COVID-19 pandemic on maternal and/or child health services. This influence was considered as reported in each included study. It was reported in quantified form (measurement with or without statistical tests). Influence also referred to any explanation (how, why) of the influence/effect of the COVID-19 pandemic on maternal and/or child health services.
Context	Sub-Saharan Africa, as defined by the United Nations Development Programme, including 46 countries, listed by region: °*East Africa*: Tanzania, Kenya, Uganda, Rwanda, Burundi, Southern Sudan, Eritrea, Ethiopia, Madagascar, Malawi, Mozambique ° West Africa: Benin, Burkina Faso, Cape Verde, Gambia, Ghana, Guinea, Guinea-Bissau, Ivory Coast, Liberia, Mali, Mauritania, Niger, Nigeria, Senegal, Sierra Leone and Togo °*Central Africa*: Angola, Cameroon, Central African Republic, Chad, Republic of Congo, Democratic Republic of Congo, Equatorial Guinea, Gabon, and Sao Tome & Principe °*Southern Africa*: Botswana, Lesotho, Namibia, South Africa, Swaziland, Zambia and Zimbabwe °*Indian Ocean*: Mauritius, Seychelles Studies written in English or French

### 2.4 Data extraction

Data were extracted by two independent reviewers (BSC and SAT) using a pre-designed extraction matrix in Microsoft Excel (Supplementary material). Each reviewer pre-tested the extraction matrix on at least five studies; based on this test, the reviewers adapted the matrix before use. The data collected included macro data (descriptive characteristics) and micro data (analytical characteristics) from the selected studies. Macro data included name of first author, year of study publication, country of study, title, objective, study design, and study setting (rural or urban). Microdata were collected on the effects of the COVID-19 pandemic on health facility attendance, ANC services (ANC visits, high-risk pregnancy screening, SP offer, iron folic acid tablets, long-acting insecticide-treated nets, HIV counseling, HIV testing), delivery coverage in primary health facilities Postpartum care coverage (care of the mother during the first 6 weeks after delivery), postnatal care coverage (care of the child during the first 6 weeks after birth), routine child vaccination, use of services for common childhood diseases in SSA (malaria, respiratory infections, diarrheal diseases, etc.), as well as factors through which COVID-19 influenced the use, uptake, and provision of maternal or child health services.

### 2.5 Data analysis

We first described article selection and summarized the characteristics of included studies. Extracted data were synthesized descriptively. The effect of the COVID-19 pandemic on primary MCH services, as reported by the authors of the studies, was summarized and presented in tables by MCH indicator. Qualitative data explaining the effect of the pandemic on primary MCH indicators were also synthesized through thematic analysis into themes and sub-themes (20). Findings were interpreted as reported by the authors of the relevant studies. Quantitative and qualitative data were analyzed separately.

## 3 Results

### 3.1 Characteristics of the studies analyzed

**Figure 1:**
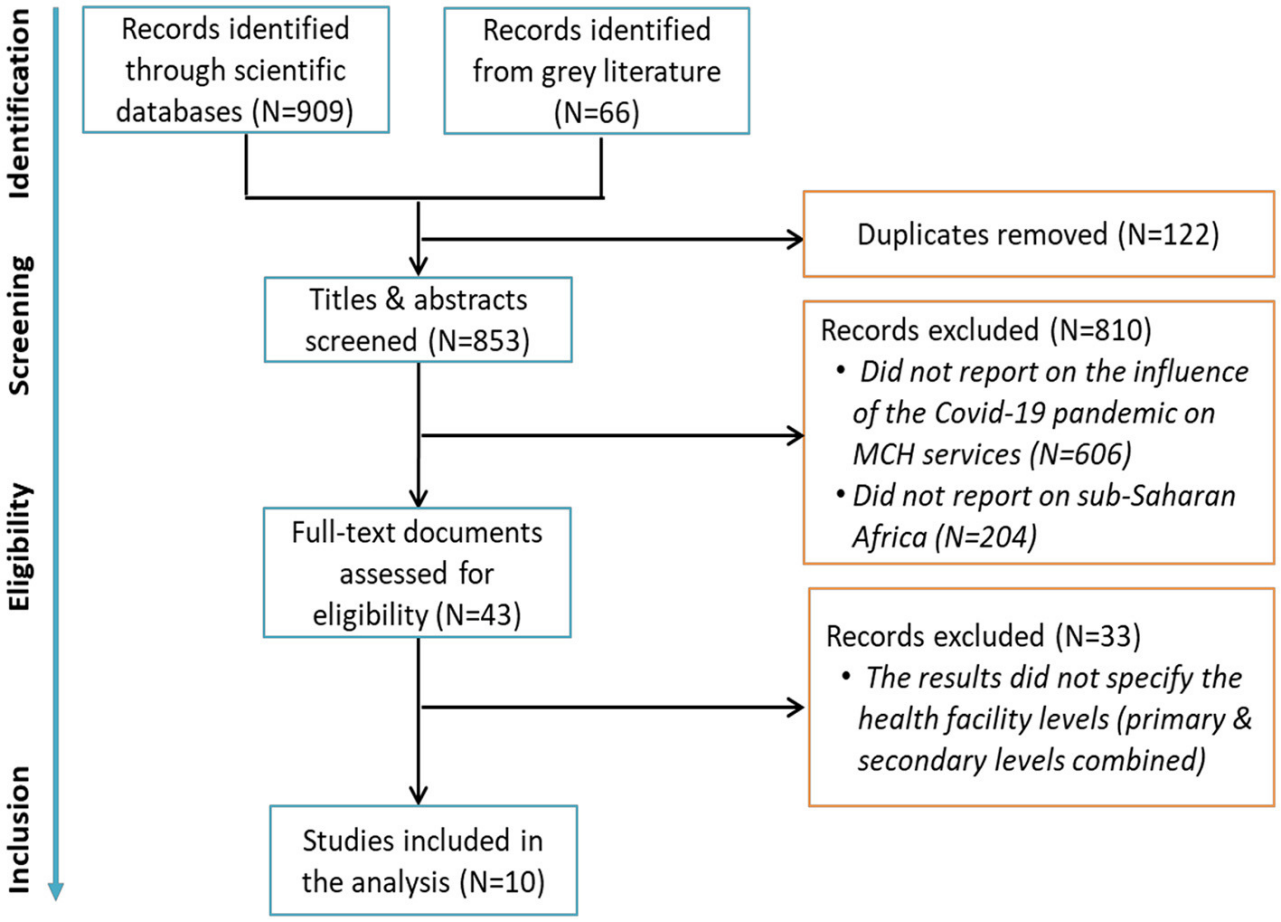
Flow chart of the review records selection.

A total of 10 studies were included in this review (Figure 1). They were conducted in six SSA countries, including Ethiopia (21–23), Ghana (24, 25), Mozambique (26, 27), Nigeria (28), Guinea (29) and Niger (30) (Table 2). These studies were published between 2021 and 2023; they included four qualitative (23, 24, 26, 28), three cross-sectional quantitative (22, 29, 30), and three mixed-methods (21, 25, 27).

**Table 2:** Characteristics of studies included in the analysis.

**Authors**	**Year of publication**	**Country**	**Area**	**Pandemic period**	**Study design**
Heuschen et al. (25)	2023	Kenya	Rural and an urban	April – May 2022	Cross sectional mixed methods
Akaba et al. (28)	2022	Nigeria	[Not reported]	May 2020	Qualitative methods
Bliznashka et al. (24)	2022	Mozambique	Rural	September – October 2020	Qualitative methods
Ndong Ignatius et al. (24)	2022	Ghana	[Not reported]	July – August 2020	Qualitative methods
Kouyate et al. (29)	2022	Guinea	Rural and Urban	March 2020 to March 2021	Retrospective cross-sectional study
Hailemariam et al. (23)	2021	Ethiopia	Urban and rural	November 2020	Qualitative methods
Mariama Baissa et al. (30)	2021	Niger	Urban	January – June 2020	Retrospective cross-sectional study
Henrique et al. (27)	2021	Mozambique	(Not reported)	March – May 2020	Cross sectional mixed methods
Workicho et al. (21)	2021	Ethiopia	Rural	March – July 2020	Cross sectional mixed methods
Ayele et al. (22)	2021	Ethiopia	(Not reported)	November 2019 – June 2020	Retrospective cross-sectional study

### 3.2 Influence of the pandemic on the use of primary MCH services

The indicators of MCH services use provided in this review, along with the corresponding studies, are presented in Table 3 and summarized below:

**Table 3:** Indicators reported and number of corresponding studies.

**Indicators reported**	**Number of studies**	**References**
1 ANC visit	3	(22, 27, 29)
4 or more ANC visits	3	(22, 27, 29)
Any ANC visits	1	(30)
Facility childbirth	2	(22, 29)
Postpartum visits	1	(27)
Pentavalent vaccine uptake	2	(22, 30)
Measles vaccine uptake	1	(30)
Overall childhood vaccine uptake	2	(27, 30)
Childhood pneumonia cases	1	(22)

ANC: antenatal care.

#### 3.2.1 Maternal health services

Four of the studies included (22, 27, 29, 30) reported COVID-19 pandemic impact on multiple maternal health service use indicators including one ANC visit [number of studies (*n*) =3], and at least four ANC visits (*n* = 3), overall number of ANC visits (*n* = 1), facility childbirth (*n* = 2), and number of postpartum visits (*n* = 1).

##### 3.2.1.1 Antenatal care

Attendance at health centers for any ANC was differentially affected by the COVID-19 pandemic as reported by one of the four studies, which showed a decrease in ANC visits in ten out of 17 health centers in Niamey (Niger); this decrease ranged in total 131 to 2,191 visits per health center in the second quarter of 2020 compared to the same period in 2019 (*p*-values not reported) (30) (Supplementary Table 1). However, the other seven health centers recorded up to a 30% increase in the number of women consulting for ANC during the second quarter of 2020 compared to the same period in 2019 (*p*-values not reported) (30).

Women's achievement of at least one ANC visit (ANC1) was reduced in Guinea (29), Mozambique (27) and Ethiopia (22). In Guinea, they decreased by 702 (95% CI = −885 to −520; *p* = 0.001) women over the first month of the pandemic (March 2020), compared to the pre-pandemic (March 2019-February 2020) monthly mean number in associative health centers; in in public health centers, this reduction was of 64 (95% CI = −137 to 9; *p* = 0.082) women (29). In Mozambique, the September 25 health center in Nampula recorded a 4% reduction in overall ANC1 (from 4,991 to 4,783 visits) and a 12% reduction (*p*-value not reported) in ANC1 performed in the first trimester of pregnancy (from 7,514 to 6,593 visits) over the March-May 2020 period compared with the same period in 2019 (24). In Ethiopia, seven public health centers in Addis Ababa had a non-significant average monthly decrease of 1.1 visits (95% CI: −7.2 to 5.0; *p* = 0.6841) over the April-June 2020 period compared with the December 2019-February 2020 period (22).

The number of women achieving at four or more ANC visits (ANC4+) decreased significantly by 1.015 (95% CI: −1.146 to −883; *p* = 0.001) on average in associative health centers in Guinea in March 2020 as compared to the monthly average number of the period March 2019-February 2020, with a reduction of; such reduction was of 794 (95% CI: −909 to −678; *p* = 0.001) in public health centers (29); nonetheless, it more than doubled (125% increase) in the September 25 health center in Nampula (24) over the March-May 2020 period compared with the same period in 2019. In Ethiopia, the decrease was non-significant in Addis Ababa health centers, with an average monthly number of 4.6 visits (95% CI: −20.3 to 11.2; *p* = 0.5233) over the April-June 2020 period compared with the December 2019-February 2020 period (22).

##### 3.2.1.2 Facility childbirth

With regard to childbirths in the health centers, the influence of the pandemic was mixed; the number of childbirths significantly decreased in the associative health centers in Guinea by a monthly average of 596 (95% CI: −677 to −516; *p* = 0.001) cases in March 2020 as compared to the March 2019-February 2020 (29). Differences identified in public health centers in Guinea (increase of a monthly average of 36 childbirths; 95% CI: −56 to 133; *p* = 0.413) (29) and in health centers in Addis Ababa, Ethiopia (increase of a monthly average of 4 childbirths; 95% CI: −1. 5 to 9.5; *p* = 0.1347) (22) were not statistically significant.

##### 3.2.1.3 Postpartum care

The influence of the pandemic on the number of women consulting for postpartum care was also reported for the September 25 health center in Nampula, Mozambique, with a statistically non-significant reduction in the monthly average number of women (1%) between March-May 2020 compared to the same period in 2019 (27).

#### 3.2.2 Child health services

Three of the studies included in this review (22, 27, 30) reported the effect of the COVID-19 pandemic on child health indicators (Supplementary Table 1). These indicators included vaccine uptake, notably uptake of overall childhood vaccine (27, 30), uptake of pentavalent vaccine (22, 30) and of measles vaccine (30); one of these studies (22) reported the number of childhood pneumonia cases seen in health centers.

##### 3.2.2.1 Vaccine uptake

The reported effect of the COVID-19 pandemic on childhood vaccine uptake varied across studies included. A non-significant reduction in the number of children fully vaccinated was reported at the Marrere health center in Natikiri (18% reduction; *p* = 0.544) and at the 25 September health center in Nampula (16% reduction; *p*-value not reported) during the period March-May 2020, compared with the same period in 2019 (27). The number of children who received at least one dose of any vaccine decreased by 20% (*p* = 0.197) and 18% (*p*-value not reported) in these health centers and over similar periods, respectively (27). However, in Niamey, Niger, while health centers of Niamey 4 health district reported no change in the number of children who received any vaccine doses in March-May 2020 as compared to the same period in 2019, health centers in Niamey 2 and Niamey 5 health districts reported more than 80% reduction (*p*-value not reported) of this number over a similar period (30).

For pentavalent vaccine uptake, declines of 49% (95% CI: −58% to −40%) in the number of children vaccinated with pentavalent 1 and 48% (95% CI: −57% to −39%) in the number of children vaccinated with pentavalent 3 were observed in health centers in Niamey, Niger, over the period January-June 2020, compared with the same period in 2019 (30). In health centers in Addis Ababa (Ethiopia) declines, although not statistically significant, were also reported in pentavalent vaccine uptake, with an average monthly drop of 4.9 children (95% CI: −3.8 to 13.6; *p* = 0.2320) for pentavalent 1 and one child (-9.6, 7.6; *p* = 0.7498) for pentavalent 3 over the period April-June 2020 compared with December 2019-February 2020 (22).

The one study that reported on measles vaccine uptake showed a significantly 35% (95% CI: −44% to −26%) drop in the number of children who received a dose of this vaccine in Niamey health centers over the period March-May 2020 as compared to the same period in 2019 (30).

##### 3.2.2.2 Childhood pneumonia cases seen in health centers

Only one study (22) reported on childhood diseases seen at heath center, specifically on childhood pneumonia cases. It showed that in Addis-Ababa, pneumonia cases seen in health centers dropped by a monthly average of 22.6 cases (95% CI: −43.9, to −1.2; *p* = 0.0407) over the period April-June 2020 compared to December 2019-February 2020 (22) (Supplementary Table 1).

### 3.3 How did the pandemic affect the use of primary MCH services?

**Figure 2:**
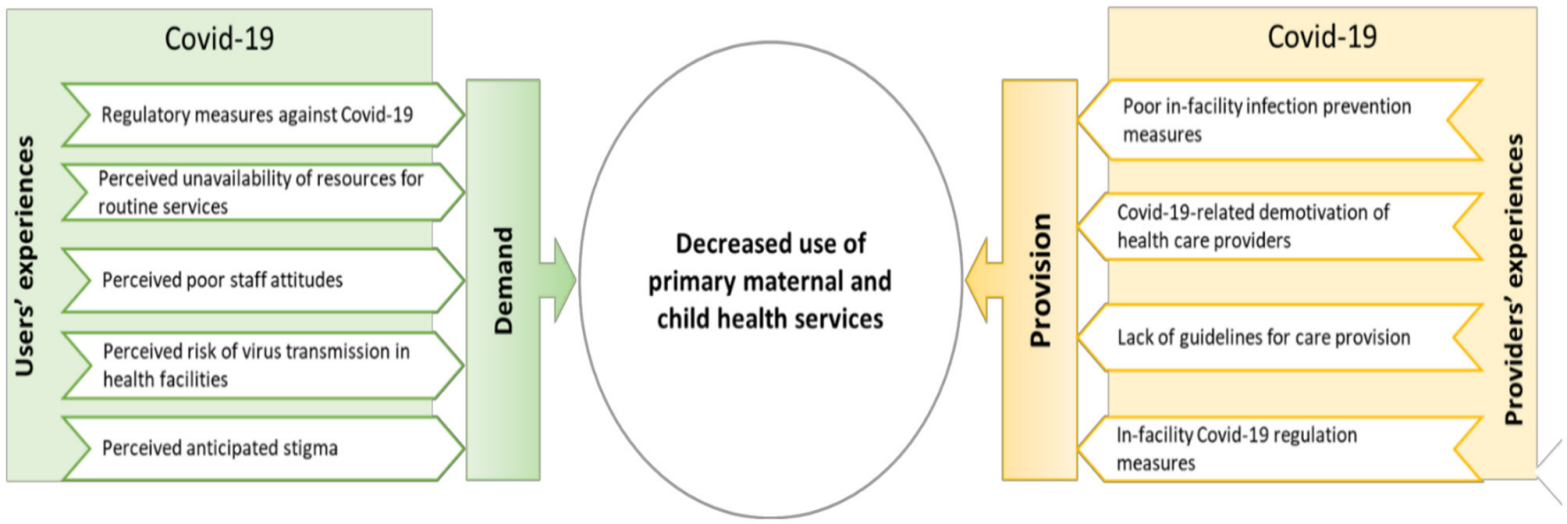
Explanatory framework for the negative influence of the COVID-19 pandemic on the use of primary maternal and child health services in sub-Saharan Africa.

Qualitative data reported by seven studies (21, 23–28) synthesized the experiences of users and providers of primary MCH services, explaining how the COVID-19 pandemic hindered the use of these services in SSA (Figure 2). These experiences were from five countries, namely Ethiopia (21, 23), Kenya (25), Nigeria (28), Ghana (24) and Mozambique (26, 27).

#### 3.3.1 Users' experiences

Five studies (21, 23, 24, 27, 28) reported on users' experiences of MCH services during the COVID-19 pandemic. These experiences revealed five main factors that hindered women's use of MCH services during the pandemic, including regulatory measures against COVID-19 (23, 27, 28), the perceived unavailability of resources in these facilities (23), the perceived poor staff attitudes (23, 24) the perceived risk of transmission of the virus in primary health care facilities (21, 23, 27, 28), and perceived anticipated stigma related to COVID-19 (23) (Supplementary Table 2).

##### 3.3.1.1 Regulatory measures against COVID-19

Government regulatory measures against COVID-19, including population lockdowns and restrictions on the number of passengers per vehicle for transportation, disrupted women's and children's use of primary health care services (23, 26, 28). During COVID-19 lockdown, the cost of living became more expensive, and parents described challenges surviving from day to day because the lockdown prevented them from engaging in their usual money-making activities (28). Furthermore, in a number of SSA countries, where transportation is a major challenge, public transport vehicles (taxis) are one of the main ways for people to get around, with several passengers (up to six) on board the same vehicle. Restrictions on the number of passengers for public transport not only worsened vehicle unavailability, but also led to an increase in domestic transport costs, preventing parents from going to the health centers for lack of transport costs (23).

Regulatory measures were also adopted in health facilities and led some users of primary MCH services to avoid using them in Ethiopia and Nigeria (23, 28). One study reported the wearing of masks was mandatory in several health centers. For some users with very low incomes, the financial limitations of purchasing a mask for each visit reduced their attendance at these facilities (28); for others, especially pregnant women, the respiratory discomfort caused by the mask led them not to want to use it, and therefore not to go to the HC for routine visits (23). As a physical distancing measure, some primary health care facilities which usually received large numbers of women and children for ANC and immunization reduced the number of patients in waiting, consultation, and treatment rooms (23, 27, 28). In addition, the number of health workers was reduced in the same setting; instead of working simultaneously, they worked in rotation (27, 28). This reorganization reduced and delayed provision of overall care, resulting in fewer patients receiving care per day and discouraging some patients who found it difficult to wait for long periods of time or to return the next day for care (23, 27, 28).

##### 3.3.1.2 Perceived unavailability of resources for routine services

The refusal of some users to use MCH services in primary health care facilities during the COVID-19 pandemic was related to their perception that health care workers were more involved in COVID-19 control activities than in routine services such as ANC, as governments prioritized the COVID-19 crisis over routine services (23). In the same vein, some users had the feeling that equipment and consumables for routine care of pregnant women and children were lacking in favor of the pandemic. (23).

##### 3.3.1.3 Perceived poor staff attitudes

Certain perceived staff attitudes, including disrespect (23) and conspiracy to deliberately transmit coronavirus (24) have been reported to have inhibited some clients' use of primary MCH services. In Ethiopia, rumors that providers during the COVID-19 pandemic crisis were much more disrespectful to pregnant women in health centers negatively influenced some pregnant women to not seek care (23). In Ghana, some pregnant women and parents refused to use health services because of rumors that health workers, in complicity with sponsors, were infecting health service users through the needle prick during the malaria rapid diagnostic test (RDT), or through the face masks they distributed (24).

##### 3.3.1.4 Perceived risk of virus transmission in health facilities

Another factor that led women and parents to turn away from primary health care facilities was the fact that they perceived these facilities as environments that were conducive to the transmission of the coronavirus in the sense that care was provided without appropriate infection prevention measures. Indeed, some users reported the inappropriate use or non-use of personal protective equipment such as masks, gloves or hydro-alcoholic gels (21, 23, 27). In addition, other users felt that the large number of people visiting these primary facilities created crowds and consequently increased the risk of transmission of the virus (21, 27, 28).

##### 3.3.1.5 Perceived anticipated stigma

In Ethiopia, users of maternal health services explained their refusal to use these services by their anxiety that they would be stigmatized by members of their communities once they visited the health facility (23). This stigma is thought to be related to the perception that people visiting health facilities are likely to carry the virus into the community. Other users – as for the same study – were reluctant to go to health facilities for fear of being labeled positive for the coronavirus and quarantined by or through the health workers' alert.

#### 3.3.2 Providers' experiences

All seven studies – in Ethiopia, Mozambique, Ghana, Nigeria – reporting qualitative data (21, 23–28) described users' experiences of factors that inhibited the provision of MCH services. These factors included poor in-facility infection prevention measures (21, 23–26, 28), COVID-19-related de-motivation of health care providers (23, 28), the lack of COVID-19-related guidelines for provision of routine care (28) and in-facility COVID-19 regulation measures (23, 25–28).

##### 3.3.2.1 Poor in-facility infection prevention measures

The disruption of primary MCH services during the COVID-19 pandemic was related to the lack of personal protective equipment for providers against coronavirus transmission (21, 23, 25, 28). This deficit led health care providers either to desert these facilities or not to provide all necessary care to patients for fear of being infected (21, 23, 26). In some cases, the inability of health centers to screen for COVID-19 – due to lack of testing – led some providers to deny care altogether to patients they suspected of having COVID-19 (24, 25, 28).

##### 3.3.2.2 COVID-19-related demotivation of health care providers

Some primary health services providers had deserted their health facilities due to demotivation; for them, providing care in a context where infection control measures are weak constitutes a great risk for providers, and therefore they felt the risk of working without the payment of risk allowances, was not worth the potential consequences (23, 28).

##### 3.3.2.3 Lack of guidelines for care provision

One study in Nigeria (28) reported the lack of COVID-19-related protocols in health facilities – including primary facilities – to guide providers in offering routine services as a challenge in providing these services.

##### 3.3.2.4 In-facility COVID-19 regulation measures

Another explanation for the disruption of service provision relates to regulatory measures adopted in health facilities to observe physical distancing. These measures consisted of reducing the number of providers on duty per day (25, 27, 28) and limiting the number of patients seen per day in order to limit caregiver-patient and patient-patient contact (23, 26, 28). This has resulted in a decrease in the provision of services to pregnant women and children attending health centers.

## 4 Discussion

The influence of the COVID-19 pandemic on primary MCH services was mixed in SSA. Attendance at some health centers declined for curative care (30), emergency care (22), ANC (29, 30), deliveries (29), immunization (30) and for pneumonia cases (22). Other health centers did not experience a significant influence of the pandemic on some of these services (22, 27, 29, 30). Moreover, ANC increased in a number of health centers (27, 30). The deterioration of indicators was linked on the demand side to regulatory measures against COVID-19 (23, 27, 28), the perceived risk of transmission of the virus in primary health care facilities (21, 23, 27, 28), the perceived unavailability of resources in these facilities (23), the perceived attitude of the staff of these facilities (23, 24) and the anxiety of the users of services (23). On the provision side, these were the lack of equipment in the primary facilities (21, 23–26, 28), the lack of guidelines for the provision of care in the context of the pandemic for these facilities (26, 28), the regulatory measures against COVID-19 that were implemented (23, 25–28) and the demotivation of the providers who worked there (23, 28).

The COVID-19 pandemic had mixed effects on MCH primary service indicators in SSA; these effects actually varied from country to country and from health center to health center within the same country. Guinea and Ethiopia were marked by declines in coverage and unchanged indicators (22, 29); Niger by declines, unchanged indicators, and increases in coverage (30); and in Mozambique, reported indicators were either unchanged or increasing during the pandemic (27). As for the intra-country variation between the health centers, it was illustrated in Guinea by a decrease in ANC1 visits in the associative health centers as opposed to the public health centers, and in Niger by a decrease in ANC visits in a selection of health centers as opposed to an increase in the others, and by a decrease in immunization in some health centers as opposed to others. The differential influence of the COVID-19 pandemic on primary MCH indicators in SSA calls into question and challenges the stereotypical homogenization of the vulnerability of SSA countries' health systems to epidemic crises (31). Indeed, the generalized judgment that these health systems are not resilient masks their individual realities; this, in turn, not only affects the prioritization of countries that really need support, but also conceals the singular experiences of success of certain health systems that could serve as examples for others to achieve resilience. The resilience of a healthcare system in the face of a COVID-19 pandemic is defined by its ability to adapt in the face of the pandemic crisis so as to retain the same control over its structure and functions (32). It is therefore worthwhile for countries whose use of primary MCH services has fallen significantly to learn from the adaptation experiences of countries whose services have not been affected.

The various indicators of MCH primary service use reported by the reviewed studies declined during the COVID-19 pandemic. Various studies have reported a similar influence in the context of this pandemic (22, 33–35). This decline was sufficiently explained by different studies analyzed in this review (21, 23–28). The various factors explaining this decline are worth discussion; however, among them, we pay particular attention here to the regulatory measures taken against COVID-19 and the lack of adequate physical resources for the safe provision of services. Different studies (23, 27, 28) have revealed that the regulatory measures taken by governments and health facilities against COVID-19 contributed to depriving many pregnant women and children from the essential health care they needed during the COVID-19 pandemic. This deprivation, which led to maternal and infant deaths (36) calls policy makers to question the adverse effect of health policies in times of crisis or health emergency on MCH indicators in SSA. This finding should be an invitation to these decision-makers to contextualize, for possible future epidemic crises in this region, the adaptive measures to be taken rather than importing locally inappropriate or damaging measures as was the case during the COVID-19 pandemic (37). For example, several SSA countries, copying the Western model of response, adopted, among other measures, containment, limiting the number of people in transport and public places in a context where the majority of households are surviving on a daily basis and where access to routine health services was already limited.

The deficiency of material resources in primary health care facilities has forced both users and providers of MCH services to avoid attending these facilities for fear of contracting the coronavirus. Various studies have already reported in different epidemic contexts the deficiency of material resources in health facilities as a factor in the underutilization of health services (35–37). Nevertheless, it is important to emphasize that this lack of material resources is more pronounced in primary than in secondary or tertiary facilities. This discrepancy is said to have worsened in the context of the COVID-19 pandemic; for example, in Guinea, a study reported that during the first months of the COVID-19 pandemic, more hospitals were supplied with protective equipment for personnel than health centers and health posts (38). Yet, in terms of health systems in SSA, primary health care facilities are the ones that provide the bulk of health care to pregnant women, mothers and children. Because of their lack of material resources, primary healthcare facilities are thus one of the main weak points in the resilience of MCH services in SSA. It is therefore crucial, in view of the recurrent threat of epidemics on this continent, to prioritize the improvement of preventive measures in primary health care facilities. This would make a significant contribution to the resilience of healthcare systems to epidemic crises in SSA.

This study had three main limitations. The first was the scarcity of eligible studies, i.e., studies that focused on primary MCH services. This scarcity did not allow for a comprehensive analysis, i.e., taking into account the contexts of most countries in SSA. It is therefore important to recommend that researchers evaluating the influence of health crises, including epidemics, on routine health services place particular emphasis on primary services. The second limitation of this study is methodological; being a scoping review, unlike the systematic review, it did not take into account the methods used by the studies to measure the effect of the pandemic on MCH indicators. Nevertheless, given the limited number of studies eligible for this review, the scoping review was the most appropriate, as a systematic review would require a sufficient number of studies (39). Lastly, this review considered the effects of the pandemic on MCH indicators as reported by the different authors of the included studies; however, some effects were reported without statistical measures. Thus, we recommend that authors of future research fully report statistical information about the effect being evaluated, including the number of observations, proportion, mean or median, effect measure (e.g., odds ratio or risk difference), and probability value. Of course, the strengths of this review include its scoping approach, which did not limit our exploration to any one type of study or source of information. In addition, it has the merit of covering different geographical (West Africa, East Africa) and cultural (Francophone, Anglophone and Lusophone countries) contexts.

## 5 Conclusion

The influence of the COVID-19 pandemic on primary MCH services is mixed in SSA. They show that attendance at some health centers declined for various services, remained unchanged or increased for other health centers. In addition, this mixed effect is marked by cross-country variation, with Mozambique showing resilience to the pandemic.

The decline in the use of health centers is explained by factors related to the pandemic, which affected the demand for and provision of services in these facilities. The factors that affected the demand for services relate to the regulatory measures against COVID-19, the perceived risk of transmission of the virus in primary health care facilities, the perceived unavailability of resources in these facilities, the perceived attitude of the staff in these facilities, and the anxiety of service users. Factors that disrupted service provision included the insufficient availability of equipment in primary health care facilities, the lack of pandemic care guidelines for these facilities, regulatory measures against COVID-19 in these facilities, and the de-motivation of providers who worked in these facilities during the pandemic.

This review discusses the need for policymakers to contextualize adaptive measures for future epidemic crises in SSA. It also recommends prioritizing the improvement of prevention measures in primary health care facilities and calls on other countries in SSA to learn from the experiences of the Mozambican health system to ensure that MCH indicators are resilient to epidemic crises.

## Author contributions

BC: Conceptualization, Data curation, Formal analysis, Investigation, Methodology, Validation, Writing – original draft. AE: Conceptualization, Formal analysis, Supervision, Validation, Writing – review & editing. AT: Data curation, Validation, Writing – review & editing. FT: Methodology, Validation, Writing – review & editing. ED: Methodology, Validation, Writing – review & editing. MD: Data curation, Methodology, Validation, Writing – review & editing. J-BL: Data curation, Validation, Writing – review & editing. MT: Validation, Writing – review & editing. AD: Conceptualization, Methodology, Supervision, Validation, Writing – review & editing.
