# Supplementary material for: Mixed influence of COVID-19 on primary maternal and child health services in sub-Saharan Africa: a scoping review
Source: Front Public Health. 2024 Jun 24;12:1399398. doi: 10.3389/fpubh.2024.1399398 (PMC11228267; doi:10.3389/fpubh.2024.1399398)
Supplement: Supplementary file 1 [file Table_1.pdf]

Supplementary Table 1: Influence of the COVID-19 pandemic on utilization of primary maternal and child health services in sub-Saharan Africa

Author, year, Country	ANC visits	Facility delivery	Postpartum care	Childhood vaccination	Services for other childhood diseases
Mariama Baissa al., 2021, Niger	**Decline in antenatal care visits (for 10 health centers)** in quarter 2 2020 compared to quarter 2 2019.<br>• Varied from -131 to -2191 women from a health center to another (for 10 health centers) [p value not reported]<br>**Increase in antenatal care visits (for 7 health centers)** in quarter 2 2020 compared to quarter 2 2019.<br>• Up to 30% [p value not reported]	[Not reported]	[Not reported]	**Decline in vaccination**<br>• -49% (95% CI: -58% to -40%) of children vaccinated with pentavalent 1 over the first 6 months of 2020, compared to 2019 [p value not reported]<br>• -48% (95% CI: -57% to -39%) for pentavalent 2 over the first 6 months of 2020, compared to 2019 [p value not reported]<br>• - 35% (95% CI: -44% to -26%) for measles vaccine over the first 6 months of 2020, compared to 2019<br>-80% in health centers of Niamey districts 2 and 5 in quarter 2 2020 compared to quarter 2 2019. [p value not reported]	[Not reported]

Ayele et al., 2021, Ethiopia	**Decline in antenatal care visits** in April-June 2020 compared to December 2019 - February 2020<br><br>• -1.1 visits (95% CI: -7.2 to 5.0; p=0,6841) on average per health center for ANC1<br>• -4.6 visits (95% CI: -20.3 to 11.2; p= 0.5233) on average per health center for ANC4	**Increase in facility deliveries** in April-June 2020 compared to December 2019 - February 2020<br><br>• 4 health center-based deliveries (95% CI: -1.5 to 9.5; p=0.1347) on average per health center	[Not reported]	**Decline in vaccination** in April-June 2020 compared to December 2019 - February 2020<br><br>• -4,9 children (95% CI: -3.8 to 13.6; p=0.2320) on average per health center for pentavalent 1<br>• -1 child (95% IC: -9.6 to 7.6; p= 0.7498) on average per health center for pentavalent 4	**Decline in pneumonia cases presenting at health centers** in April-June 2020 compared to December 2019 - February 2020<br><br>• -22.6 cases (95% IC: -43.9 to 1.2; p=0.0407) on average per health center
Kouyaté et al., 2021, Guinea	**Decline in antenatal care visits** in March 2020 as compared to the monthly mean number of the March 2019 – February 2020 period<br><br>• -702 women (95% CI: -885 to -520; p = 0.001) attending ANC1 in associative health centers<br>• -64 women (95% CI: -137 to 9; p = 0.082) attending ANC1 in public health centers<br>• β = −1.015 (95% CI: -1.146 à -883; p = 0.001) as immediate effect for ANC4 in associative health centers	**Decline in facility deliveries for associative health centers** in March 2020 as compared to the monthly mean number of the March 2019 – February 2020 period<br><br>• -596 women (95% CI: -677 to -516 ; p = 0.001) giving birth in health centers<br><br>**Increase in facility deliveries for public health centers** in March 2020 as compared to the	[Not reported]	[Not reported]	[Not reported]

	• β= −794 (95% CI: -909 to -678 ; p = 0.001) as immediate effect for ANC4 in public health centers	monthly mean number of the March 2019 – February 2020 period<br><br>• 36 women (95% CI: -56 à 133 ; p = 0.413) giving birth in health centers			
Henrique et al., 2021, Mozambique	**Decline in antenatal care visits (1st visits)** in March-May 2020 compared to March-May 2019:<br><br>• -4% [p value not reported]<br>• -12% [p value not reported] for ANC 1 occurring over the 1st trimester of pregnancy<br><br>**Increase in antenatal care visits (4th visits)** in March-May 2020 compared to March-May 2019:<br><br>• 125% [p value not reported]	[Not reported]	**Decline in postpartum visits** in March-May 2020 compared to March-May 2019<br><br>• -1% (from 2795 to 2761) [p value not reported]	**Decline in vaccination** in March-May 2020 compared to March-May 2019<br><br>• -2% [p value not reported] of children who received at least a vaccine dose in 25 September health center<br>• -20% (p=0.197) of children who received at least a vaccine dose in Marrere health center<br>• -16% [p value not reported] of children who received complete vaccine doses in 25 September health center<br>• -18% (p=0.544) of children who received complete vaccine doses in Marrere health center	[Not reported]
