# Supplementary material for: Mixed influence of COVID-19 on primary maternal and child health services in sub-Saharan Africa: a scoping review
Source: Front Public Health. 2024 Jun 24;12:1399398. doi: 10.3389/fpubh.2024.1399398 (PMC11228267; doi:10.3389/fpubh.2024.1399398)
Supplement: Supplementary file 2 [file Table_2.pdf]

Supplementary Table 2: Key themes/sub-themes and selected quotations from qualitative/mixed-methods studies included in the review, explaining the factors that hindered use of maternal and child health services in sub-Saharan Africa during the COVID-19 pandemic

	Themes	Sub-themes	Supporting quotes
**Users' experiences**	Regulatory measures against COVID-19	Population lockdowns (28)	*"...if I no go market, ... how can I get money to go that antenatal",* Antenatal care service user, Nigeria (28)
		Restrictions on the number of passengers per vehicle for transportation (23)	*"Now, transportation cost is doubled. For this reason, I am forced to pay for two seats. ...It is difficult for me to afford all those things where my income is decreased by the pandemic already."* Antenatal care service user, Ethiopia (23)
		Financial limitations to purchase face mask (28)	*"The instruction from the state is that no face mask no entry, so it has really been difficult to really assess patients that need our services once they cannot afford the hundred naira face mask, they turn them out, they can't enter the hospital."* Policy Maker, Secondary Health Centre, Nigeria
		Perceived discomfort to use face mask (23)	*"It is mandatory for every individual to use a face mask, but when I use a face mask, ... I can't talk and breathe freely. I also feel suffocated and my breath smells bad. For this reason, I often chose to stay home."* Antenatal care service user, Ethiopia (23)

	Limitation of patients' number for physical distancing (23,27,28)	*…You get to the hospital around 7am, you'd have to leave around 12pm. I think the doctors there are not enough, we'd have to sit and sit and sit, you know. It's time consuming. That's the only challenge I have oh! if you gather as early as 7:30am, 6:30am but you'd have to stay till 12:00pm sometimes, or 2:00pm which is very frustrating.* Antenatal care service user, Nigeria (28)
Perceived unavailability of resources for routine services	Health care workers more involved in COVID-19 control at the expenses of routine services (23)	*"These days everyone is talking about coronavirus, and I don't think that healthcare providers have a time to treat pregnant women as usual. Thus, what is the point of visiting a health facility for antenatal care if you don't have enough time to be treated and advised?"* Antenatal service user, Ethiopia (23)
	Perceived lack of equipment/consumables for routine care in favor of the pandemic (23)	*"I don't think the health care facilities in this pandemic period have the necessary materials for providing antenatal care service. ...the Medias, the government, and everybody is saying corona, corona, corona ..."* Antenatal service user, Ethiopia (23)
Perceived poor staff attitudes	Disrespect (23)	*"I would not advice pregnant women to visit a health facility during this corona virus period. What I heard from those who visit a health facility is completely discouraging; health care providers often disgrace you and even insult you."* Antenatal service user, Ethiopia (23)
	Conspiracy to deliberately transmit coronavirus (24)	*« We heard that the nasal masks could be infected with the virus and we will get sick. They said that if we allow you to prick us, we will get the disease»* Child health service user, Ghana (24)

	Perceived risk of virus transmission in health facilities	Inappropriate/non-use of personal protective equipment (21,23,27)	*"As my neighbor told me, healthcare providers often use the same glove for different clients, and they don't use alcohol regularly; ...Some of them even move here and there but they don't change their gloves before toughing you."* Antenatal service user, Ethiopia (23)
		Overcrowding of health facilities (21,27,28)	*"Health facilities give service for all clients coming from different areas; this results in overcrowding and makes it easier for corona transmission..."* Child health service user, Ethiopia (23)
	Perceived anticipated stigma	Stigma that people attending health facility carry coronavirus into the community (23)	*"I have witnessed that women who visit a health facility for any reason were considered to bring the virus into the community; thus, people refrain from meeting them."* Antenatal service user, Ethiopia (23)
		Fear of being labeled positive for the coronavirus (23)	*"I am afraid of visiting a health facility in this pandemic period because health care providers test every one coming to the clinic and who knows. . .I may end up in quarantine just because my body become hot due to reasons other than corona. If so, who would care for my children, while I'm in quarantine for about 2 weeks?"* Antenatal service user, Ethiopia (23)
**Providers' experiences**	Poor in-facility infection prevention measures	Lack of personal protective equipment for providers (21,23,25,28)	*"Since the Corona virus pandemic, we have been facing a severe shortage of essential drugs and supplies such as alcohol, iron, face masks and other personal protective equipment…. To reduce the risk of transmission, we usually compromise routine prenatal care. For example, we may not perform a physical examination or blood test, even if it is necessary."* Maternal health service provider, Ethiopia (23)

	Inability of health centers to screen COVID-19 cases (24,25,28)	*"This child was sick and when we came [to the health center], they refused to take care of him because of the high temperature he had."* Child health service user, Ghana (24)
COVID-19-related demotivation of health care providers	COVID-19-related demotivation of health care providers (23,28)	*"After working all day in the workplace, I go home at night; imagine the risk I could put my family at. Why would I take such a risk? When the government is not even willing to pay hazard pay...I don't want to work in this environment anymore."* Maternal health service provider, Ethiopia (23)
Lack of COVID-19-related guidelines for provision of routine care	Lack of COVID-19-related guidelines for provision of routine care (26,28)	[No quote reported for primary health facility]
In-facility COVID-19 regulation measures	Limiting the number of providers per day (25,27,28)	*There are just two or three personnel that you will see to attend to the large number of women.* – Antenatal health service user, Nigeria (28)
	Limiting the number of patients per day (23,26,28)	*"We wanted to stick to twenty patients a day, because of the social distancing and we wanted to avoid overcrowding. So we only deal with twenty clients a day, yes, twenty a day."* Maternal health service provider, Nigeria (28)
